# A mitogenomic phylogeny of chitons (Mollusca: Polyplacophora)

**DOI:** 10.1186/s12862-019-1573-2

**Published:** 2020-02-05

**Authors:** Iker Irisarri, Juan E. Uribe, Douglas J. Eernisse, Rafael Zardoya

**Affiliations:** 1 Department of Biodiversity and Evolutionary Biology, Museo Nacional de Ciencias Naturales (MNCN-CSIC), c/ José Gutiérrez Abascal 2, 28006 Madrid, Spain; 2 Department of Organismal Biology (Systematic Biology Program), Evolutionary Biology Centre, Uppsala University, Norbyv. 18C, 75236 Uppsala, Sweden; 3 Department of Invertebrate Zoology, Smithsonian Institution, National Museum of Natural History, 10th St. & Constitutional Ave. NW, Washington, DC 20560 USA; 4 Department of Biological Science, California State University Fullerton, 800 N. State College Blvd, Fullerton, CA 92831-3599 USA

**Keywords:** Bayesian, Evolution, Fossil, Maximum likelihood, Mitochondrial genome, Molecular clock, Mollusk, Mt, Timetree

## Abstract

**Background:**

Polyplacophora, or chitons, have long fascinated malacologists for their distinct and rather conserved morphology and lifestyle compared to other mollusk classes. However, key aspects of their phylogeny and evolution remain unclear due to the few morphological, molecular, or combined phylogenetic analyses, particularly those addressing the relationships among the major chiton lineages.

**Results:**

Here, we present a mitogenomic phylogeny of chitons based on 13 newly sequenced mitochondrial genomes along with eight available ones and RNAseq-derived mitochondrial sequences from four additional species. Reconstructed phylogenies largely agreed with the latest advances in chiton systematics and integrative taxonomy but we identified some conflicts that call for taxonomic revisions. Despite an overall conserved gene order in chiton mitogenomes, we described three new rearrangements that might have taxonomic utility and reconstructed the most likely scenario of gene order change in this group. Our phylogeny was time-calibrated using various fossils and relaxed molecular clocks, and the robustness of these analyses was assessed with several sensitivity analyses. The inferred ages largely agreed with previous molecular clock estimates and the fossil record, but we also noted that the ambiguities inherent to the chiton fossil record might confound molecular clock analyses.

**Conclusions:**

In light of the reconstructed time-calibrated framework, we discuss the evolution of key morphological features and call for a continued effort towards clarifying the phylogeny and evolution of chitons.

## Background

Chitons (Polyplacophora) are exclusively marine mollusks inhabiting a wide range of habitats from the intertidal zone to the deep sea. They generally display a conserved morphology with eight dorsal (usually overlapping) shell plates or valves, surrounded by a girdle that can bear ornamentations [1]. Chitons crawl with a ventral muscular foot that is surrounded by grooves containing rows of gills (ctenidia). Dorsal valve surfaces are covered with thousands of networked sensory organs (aesthetes). There is no head and the oral region lacks eyes or tentacles; chitons can taste the substratum with a tongue-like subradular organ, and scrape or bite food with a typical molluscan radula. The radula is ribbon-like with up to hundreds of rows of teeth, including a single pair per row coated with an extremely hard magnetite biomineral. In comparison to species-rich gastropods or bivalves, chitons are a relatively small group with about ~ 1000 living and 430 fossil species recognized [2, 3]. Among mollusks, living chitons are considered to be most closely related to Solenogastres (Neomeniomorpha) and Caudofoveata (Chaetodermomorpha) together forming the clade Aculifera, the sister group to Conchifera (i.e., all other living mollusks). The Aculifera hypothesis is supported by recent molecular phylogenies [4–7], paleontology [8–11], and larval musculature conditions [12]. Previously, some phylogenetic analyses proposed a closer relationship of chitons to Conchifera based on morphology (Testaria hypothesis; [13, 14], or to Monoplacophora based on molecular data (Serialia hypothesis; [15, 16]; but see [17]). The phylogenetic position of Polyplacophora holds the key to discriminating among proposed hypotheses for the mollusk phylogeny.

Chitons or chiton-like aculiferans have a long evolutionary history dating back at least to the Upper Cambrian [3, 8, 18, 19]. However, most described chiton fossils are either rather recent (Late Pliocene or younger; < 4 Ma) or are so old (i.e., Paleozoic) that the comparison to modern chitons is difficult. Despite some exceptions [20–22], older fossils, especially from the Mesozoic, are comparatively scarce and in some cases of uncertain taxonomic assignment [3]. Identifying the phylogenetic affinity of fossils is particularly challenging in chitons given the limited utility of shell characters and the difficulty of assembling the complete set of valves (most fossils are isolated valves).

According to the latest classification by Sirenko [23], modern chitons or Neoloricata (approximately corresponding to the chiton crown-group) are arranged into two orders, Lepidopleurida and Chitonida, and the latter further divided into two suborders, Chitonina and Acanthochitonina. The relatively few molecular studies across all chitons [24] or for specific groups [25–28] have generally supported these divisions but have made only limited progress in resolving the relationships among major chiton lineages and in testing thoroughly the superfamily and family arrangements as proposed by Sirenko [23]. Members of Chitonida exhibit derived valve features compared to Lepidopleurida, such as the distal extensions of the articulamentum shell layer that anchor valves into the surrounding girdle (insertion plates); these extensions are slit with rays to permit the innervation of aesthete sensory organs spread across dorsal valve surfaces [29]. Chitonida also differ from Lepidopleurida in their lateral (not posterior) gill arrangement, ornamented egg hulls, highly modified sperm acrosomes, and fertilization processes [1, 30]. Based on outgroup comparisons with other mollusks and animals, these features are considered likely derived for Chitonida whereas most key morphological characters defining Lepidopleurida have been inferred to be plesiomorphic [28, 31, 32]. Nonetheless, Lepidopleurida share at least one morphological synapomorphy: a unique sensory organ in the anterior portion of the ventral pallial groove [31]. Within Chitonida, Acanthochitonina share egg hull features and derived (abanal) gill arrangement [1, 33–35], but relationships within this group remain controversial. Likewise, relationships within the more species-rich Chitonina have remained mostly unresolved, and the family Callochitonidae has alternatively been treated as either the sister lineage to all other Chitonida [30] or nested within Chitonina [23, 36].

Mitochondrial genomes (or mitogenomes) have long been used to infer phylogenetic relationships in bilaterian animals. They are relatively easy to amplify and sequence, and provide a fair number of nucleotides (or amino acids) for robust phylogeny estimation; they have a mixture of conserved and variable sites that facilitate primer design and provide information at various divergence levels; compared to nuclear genomes, the conserved set of single-copy genes makes orthology assessment straightforward and allow virtually no missing data (the same genes are present in almost all species); mitochondrial gene products are involved in housekeeping functions that are conserved across and predate bilaterians, and thus expected to be little influenced by functional convergence [37]. In addition, the almost exclusive maternal inheritance of mitogenomes results in the absence of recombination (with few exceptions, [38]), which can mislead phylogenetic inference methods [39]. In particular, the transmission of male mitochondria is prevented in Chitonida thanks to an unusual fertilization process where sperm digests a minute pore in the egg hull and injects the male nuclear DNA but blocks entry of sperm organelles [1, 32]. In addition to sequence data, rare genomic changes such as gene rearrangements and duplications can provide additional characters of phylogenetic utility [40]. Bilaterian mitogenomes also present some drawbacks, including their relatively high substitution rate compared to nuclear genes [41, 42] that can lead to sequence saturation and long-branch attraction artifacts for deep divergences [43]. Faster evolutionary rates of rearranged genes and base compositional changes produced by gene inversions (due to mitochondrial strand biases [44];) can pose additional challenges to phylogenetic inference methods.

Despite these known analytical challenges, mitogenomes have been successfully used to reconstruct robust phylogenies in many animal groups, including mollusks (e.g., [7, 26, 45, 46]). However, the thus far seven available chiton mitogenomes only represent a small glimpse of the diversity of the group and representatives of major lineages, remarkably Lepidopleurida and Callochitonidae, have been missing. This hinders not only the inference of their overall phylogeny, but also of accurate divergence times and the evolution of their mitogenome organization. The rather conserved organization of reported chiton mitogenomes [26, 47, 48] might be beneficial for phylogenetics because it suggests limited sequence composition differences among lineages that can easily be accounted for with data partitions or mixture models.

Here, we sequenced the mitogenomes of 13 chitons across all major lineages and analyzed them together with available data from additional species in order to reconstruct a phylogeny of chitons. Using a relaxed molecular clock calibrated with fossil evidence, we inferred divergence times and assessed their robustness by sensitivity analyses under various calibration schemes, calibration density parameterizations, and clock models. We studied chiton mitochondrial gene orders and inferred the most likely scenario of gene order rearrangements. Finally, we discussed the evolution of key morphological features considering the chiton fossil record and our new time-calibrated phylogeny, and call for a continued effort towards clarifying the phylogeny and evolution of these fascinating mollusks.

## Methods

### Sequencing and assembly of mitogenomes

Information on studied species, vouchers, and sampling localities can be found in Additional file 1. Total genomic DNA was isolated using the DNeasy® Blood & Tissue Kit (QIAGEN, Carlsbad, CA, USA). The mitogenomes were amplified in a two-step procedure. First, fragments of *rrnL*, *cox1, cox3* and *cob* were amplified with universal primer pairs (see Additional file 2). PCR reactions contained 2.5 μl 10× *Taq* Buffer advanced, 1.5 μl MgCl_2_ (25 mM), 0.5 μl dNTP mixture (10 mM each), 0.5 μl of each primer (10 μM each), 0.5 μl template DNA (10–40 ng/μl), 0.2 μl 5PRIME® *Taq* DNA polymerase (5 units/μl; 5PRIME GmbH, Hamburg, Germany), and DEPC water up to 25 μl. PCR cycling conditions were as follows: initial denaturation step at 94 °C for 5 min, 45 cycles of denaturing at 94 °C for 60 s, annealing at 44–57 °C for 60 s, and extending at 72 °C for 90 s, and a final extension at 72 °C for 5 min. PCR products were purified by ethanol precipitation [49] and sequenced in automated DNA sequencers (ABI PRISM® 3700) using the BigDye® Terminator v3.1 cycle-sequencing kit (Applied Biosystems, Foster City, CA, USA) and PCR primers, following the manufacturer’s instructions.

In a second step, the obtained sequences were used to design specific primer pairs for long PCR amplification of the remaining mitochondrial genome in 2–3 overlapping fragments (see Additional file 2). Long-PCR reactions contained 2.5 μl of 10× LA Buffer II (with MgCl_2_), 4 μl dNTP mixture (2.5 mM each), 0.5 μl of each primer (10 μM), 0.5 μl template DNA (10–40 ng/μl), 0.25 μl TaKaRa LA® *Taq* DNA polymerase (5 units/μl; TaKaRa BioInc., Otsu, Japan) and DEPC water up to 25 μl. Long-PCR cycling conditions were as follows: initial denaturation step at 98 °C for 30 s; 45 cycles of denaturation at 98 °C for 10 s, annealing at 50–68 °C for 30 s, and extension at 68 °C for 60 s per Kb of PCR product, and a final extension step at 68 °C for 15 min. Long-PCR products were purified by ethanol precipitation and all fragments corresponding to each mitochondrial genome were pooled together in equimolar concentrations for further steps. The mitogenome of *Hanleyella oldroydi* (Dall, 1919) and partial mitogenomes of *Dendrochiton gothicus* (Carpenter, 1864) (6764 bp) and *Acanthochitona avicula* (Carpenter, 1857) (2600 bp) were sequenced with a shotgun protocol using the TOPO® Shotgun Subcloning Kit (Invitrogen, Carlsbad, CA, USA). Random clone libraries were constructed following the manufacturer’s instructions; briefly, PCR products were sheared into ~ 1 Kb fragments, which were end-repaired with T4 and Klenow DNA polymerases. Then, the fragments were cloned into pCR®4Blunt-TOPO® vectors and transformed into TOPO10 *E. coli* chemically competent cells. A total of 198, 161, and 114 recombinant clones were Sanger-sequenced with the universal M13 forward primer for *H. oldroyidi*, *D. gothicus* and *A. avicula*, respectively. The remaining fragments from *D. gothicus* and *A. avicula* and all other new mitogenomes were sequenced with the Illumina technology. For each species, indexed pair-end (2 × 100 bp) DNA libraries were constructed with either the TruSeq® DNA Sample Kit (HiSeq) or the Nextera XT DNA Library Prep Kit (MiSeq) (Illumina, San Diego, CA, USA), following the manufacturer’s instructions. The indexed libraries were loaded with several other indexed mitogenomes and RNAseq data from other projects into either Illumina HiSeq2000 (at Macrogen, Seoul, Korea) or Illumina MiSeq V2 500 (at Sistemas Genómicos, Valencia, Spain).

The Sanger shotgun clones were assembled using Sequencher v.5.0.1 (Gene Codes Co., Ann Arbor, MI, USA). For Illumina data, reads corresponding to different individuals were demultiplexed by the corresponding library indices and assembled using the TRUFA webserver v.0.13.3 [50]. Briefly, TRUFA performs a quality control with FastQC [51], quality-filters and trims adapters with PRINSEQ [52] and assembles contigs de novo with Trinity [53]. In a next step, Geneious® v.10.2.3 was used to extend and fuse the assembled contigs by repeatedly mapping the original filtered reads (requiring a minimum identity of 99%), and to estimate sequencing depth. Mitogenomes were annotated by similarity to available chiton mitogenomes using Geneious and later corroborated using MITOS [37], which takes into account the inferred secondary structure of transfer RNAs (tRNAs). Ribosomal RNA (rRNA) genes were assumed to extend to the boundaries of adjacent genes [54]. In the case of *Plaxiphora albida* (Blainville, 1825), the final mitogenome is a composite from two partial ones that were amplified, sequenced, and assembled independently from two conspecific individuals from nearby localities (see Additional files 1 and 2) and later merged for the final alignments. In addition to newly sequenced mitogenomes, we also annotated two then unpublished mitogenomes available in GenBank: *Acanthochitona* cf. *rubrolineata* (Lischke, 1873) (KY827039 [55];) and *Ischnochiton hakodadensis* Carpenter, 1893 (KY827038 [56];). We further assembled transcriptomes from four available chiton RNAseq datasets: *Acanthochitona crinita* (Pennant, 1777) (SRR5110525; [57]), *Leptochiton rugatus* (Carpenter in Pilsbry, 1892) (SRR1611558 [58];), *Chiton* (*Rhyssoplax*) *olivaceus* Spengler, 1797 (SRR618506 [59];), and *Tonicella lineata* (Wood, 1815) (SRR6926331 [60];). Transcriptomes were assembled de novo using Trinity v.2.8.2 and protein-coding and rRNA genes were identified by homology search against available chiton mitogenomes using BLAST [61]. Sequencing depth, length, annotation, GenBank accession numbers, and vouchers of the new mitogenomes are available in Additional file 1.

### Phylogenetic analysis

We used the new chiton mitogenomes together with those available for *Katharina tunicata* (Wood, 1815) [62], *Sypharochiton pelliserpentis* (Quoy & Gaimard, 1835) and *Sypharochiton* sinclari (Gray, 1843) [48], *Cryptochiton stellerii* (Middendorff, 1847), *Cyanoplax* cf. *caverna* (Eernisse, 1986), and *Nuttallina californica* (Reeve, 1847) [26], and *Chaetopleura apiculata* (Say, 1834) [47]. Solenogastres and Caudofoveata mitogenomes were used as outgroup [7, 45]. Ribosomal RNA genes and predicted amino acid sequences of protein-coding genes (using the invertebrate mitochondrial genetic code) were extracted from all mitogenomes. Individual proteins and rRNA genes were aligned with MUSCLE [63] as implemented in SeaView v.4.4.3 [64] and positions with > 80% gaps were trimmed off using BMGE v.1.12 [65]. Single gene alignments were concatenated into two matrices, one consisting exclusively of mitochondrial proteins and a second one additionally including rRNA nucleotide sequences. The amino acid composition of the protein matrix was studied using the χ^2^-test implemented in IQ-TREE v.1.6.10 [66] and the matched-pair tests of symmetry implemented in symtest v.2.0.37 [67].

The protein matrix was treated as a single partition. In the maximum likelihood (ML) framework, model fit was assessed in two steps: first, the best-fit replacement matrix was selected by the Bayesian Information Criterion (BIC) with ModelFinder as implemented in IQ-TREE. Then, we assessed the fit of adding empirical profile mixtures (C10 to C60 [68];), but this did not result in a better fit according to BIC (Additional file 3). Thus, the ML phylogeny was estimated with IQ-TREE under MtZoa+F + I + Γ4 and 1000 replicates of non-parametric bootstrapping to assess branch support (‘-m mtZOA+F+I+G4 -b 1000’). In the Bayesian inference (BI) framework, the relative fit of the BIC-selected site-homogeneous model (MtZoa+Γ4) was also compared to more sophisticated mixture models (MtZoa+C60 + Γ4, CAT+Γ4, and CAT-GTR + Γ4) using a 10-fold cross-validation procedure. Cross-validation analyses clearly indicated that CAT-GTR fit the data better than MtZoa+Γ4 (10 out of 10 comparisons), which was second best compared to all other models (Additional file 3). BI was performed with PhyloBayes MPI v.1.8 [69] under both CAT+GTR + Γ4 (‘-cat -gtr -dgam 4’) and MtZoa+Γ4 (‘-mtzoa -ncat 1 -dgam 4’) models. For each analysis, two independent MCMC chains were run until convergence, assessed with PhyloBayes’ built-in tools (maxdiff < 0.1 and minimum effective size > 500; Additional file 3) and Tracer v.1.7.1 [70]. The first 25% cycles were discarded as burnin.

The matrix of proteins and rRNA genes was treated as gene-partitioned, selecting best-fit models and partitions with BIC in ModelFinder as implemented in IQ-TREE and assuming edge-linked partitions (‘-m TESTMERGEONLY -spp’). The inferred best-fit models and partitions can be found in Additional file 3. Using the selected scheme (per-gene partitions), a ML tree was estimated with IQ-TREE and 1000 replicates of non-parametric bootstrapping. Two independent BI analyses were run using MrBayes v.3.2.1 [71], each with four MCMC chains for > 4 million generations. The first 25% generations were discarded as burnin and convergence was assessed *a posteriori* using Tracer, and all parameters obtained ESS > 200.

### Divergence time analyses

A total of 15 calibration points were used with minimum and maximum ages derived from the fossil record and modeled as soft bounds. To account for the uncertainty and different interpretations of the fossil record, two alternative calibrations were used each for the crown-groups Polyplacophora and Chitonida (i.e.*,* all living and extinct species descending from the most recent common ancestor of the living members) and all four possible combinations were tested in alternative calibration schemes (the remaining 13 calibration points were unaffected). Our root assumed monophyly of Polyplacophora based on previous molecular and morphological evidence [4, 8, 11]. The root (i.e., the split between crown groups Aplacophora and Polyplacophora) was calibrated at: (1a) 449.5–549 Ma based on the Ordovician *Echinochiton dufoei* Pojeta Eernise, Hoare & Henderson, 2003, which despite diverse interpretations [8, 9, 11, 72] is considered more closely related to modern chitons than to aplacophorans, or (1b) 425–549 Ma based on the Silurian *Acaenoplax hayae* Sutton, Briggs, Siveter & Sigwart, 2001, considered within the total (i.e., stem plus crown) group Aplacophora [8, 9, 11, 73]. The maximum age for the root calibrations is derived from the Cambrian deposits of the Nama group, which preserved an open marine community including the earliest animal remains but no skeletal remains of mollusks [74]. (2) Lepidopleurida was constrained at 201.3–359 Ma based on *Leptochiton* spp. fossils from the Upper Triassic [20, 75]. The maximum for this and all other subsequent calibrations was set at 359 Ma as a conservative bound based on the first appearance of modern chitons with articulamentum (Neoloricata) at the beginning of the Carboniferous [3, 23]. (3) The split between *Hanleyella oldroydi* and *Leptochiton nexus* Carpenter, 1864 was constrained at 23–359 Ma based on Oligocene fossils of the former genus [23]. For Chitonida, the oldest calibration is (4a) 174–359 Ma based on Jurassic fossils such as *Allochiton* Fucini, 1912 and *Heterochiton* Fucini, 1912 [76] and *Ischnochiton marloffsteinensis* Fiedel & Keupp, 1988 [22]. Because these fossils are much older than most other known Chitonida, the evidence for the typical Chitonida insertion plate slit rays is unclear, and they are described in single old studies, we used the alternative calibration (4b) 66–359 Ma based on the second oldest known Chitonida represented by the genus *Chiton* (see calibration 13). (5) 33.9–359 Ma for the crown-group Acanthochitonina based on *Plaxiphora* spp. and *Acanthochitona* spp. fossils [3]. (6) 33.9–359 Ma based on *Acanthochitona* spp. fossils [3] to date its split from *Hemiarthrum setulosum* Carpenter [in Dall], 1876. (7) 5.3–359 Ma based on *Acanthochitona crinita* fossils [3] to date its split from *Acanthochitona* cf. *rubrolineata*. (8) 3–359 Ma based on *Nuttallina* spp. fossils from the San Diego Formation [77] to date its split from *Cyanoplax* cf. *caverna*. (9) 15–359 Ma the family Mopaliidae, based on the earliest known fossils of *Mopalia* spp. [78]. (10) 2.6–359 Ma based on *Cryptochiton* spp. fossils [3] to date its split from *Dendrochiton gothicus,* (11) 2.6–359 Ma based on *Katharina tunicata* fossils [3] to date its split from *Tonicella lineata*. (12) 33.9–359 Ma based on fossils of *Chaetopleura apiculata* [3] to date its split from *Ischnochiton hakodadensis* [63]. (13) 66–359 Ma to date the common ancestor of *Chiton* and *Sypharochiton* based on the presence of several Late Cretaceous fossils such as *Chiton berryi* Smith, Sohl & Yochelson, 1968 [79], which also represents the oldest Chitonida after *Allochiton, Heterochiton* and *Ischnochiton marloffsteinensis*. (14) 33.9–359 Ma for the *Acanthopleura* + *Tonicia* clade based on fossils of the latter genus [3]. 0.01–359 Ma based on the *Sypharochiton pelliserpentis* fossil (=*Chiton pelliserpentis;* [3]) to date its split from *Sypharochiton sinclairi*.

Divergence time analyses relied on the Bayesian MCMCTree program within the PAML software package v.4.9e [80]. We used the protein dataset and the tree topology of BI under CAT-GTR, except that one multifurcation was resolved according to the BI tree under MtZoa (Additional file 4) because MCMCTree does not accept them. The root age was modeled using a uniform distribution, while all other calibrations were modeled using either (i) uniform distributions, (ii) truncated-cauchy (t-cauchy) distributions with long tails, or (iii) t-cauchy distributions with short tails. Compared to uniform bounds, t-cauchy aims to model the prior divergence times using probabilistic distribution where most of the prior probability is closer to the minimum age while also retaining considerable probability mass on its tail that goes back in time. The parameterizations of t-cauchy distributions followed Dos Reis et al. (i.e. *p* = 0, *c* = 0.1/10, *p*_*L*_ = 0.001) [81]. Both the uncorrelated lognormal and autocorrelated relaxed clock models were tested. Calculations relied on approximate likelihood, which uses the gradient and Hessian matrix of the likelihood at the ML estimates of branch lengths [82, 83], which were performed with CODEML (within the PAML package) under the MtZoa+Γ4 model. Priors on the mean (or ancestral) rate “rgene_gamma” were set to either G (2, 7.797) or or to G(2, 7.609) for schemes incorporating the root calibration 1a or 1b, corresponding to diffuse priors with mean rates of 0.2565 and 0.2628 amino acid replacements site^− 1^ Myr^− 1^, respectively. Mean rates were approximated using the average root-to-tip paths in the tree of Fig. 2 and mean root ages at 499 or 487 Ma (mean of maximum-minimum bounds) for schemes with calibrations 1a or 1b, respectively. The prior on the σ^2^ parameter (“sigma2_gamma”) was set to G(2,2) indicating serious violation of the strict molecular clock. The tree prior assumed a uniform birth-death process with default parameters. The time unit was set to 100 Myr. All analyses were run for two million cycles, sampling every 100, after the initial 20,000 cycles that were discarded as burnin. Each analysis was run twice to ensure convergence, which was checked *a posteriori* in Tracer v.1.5. All runs showed good convergence and ESS values > 200. In total, 48 MCMC chains were run (four calibration schemes, three calibration distributions, two clock models, two chains per setting combination).

## Results

### Mitochondrial genome organization

**Fig. 1:**
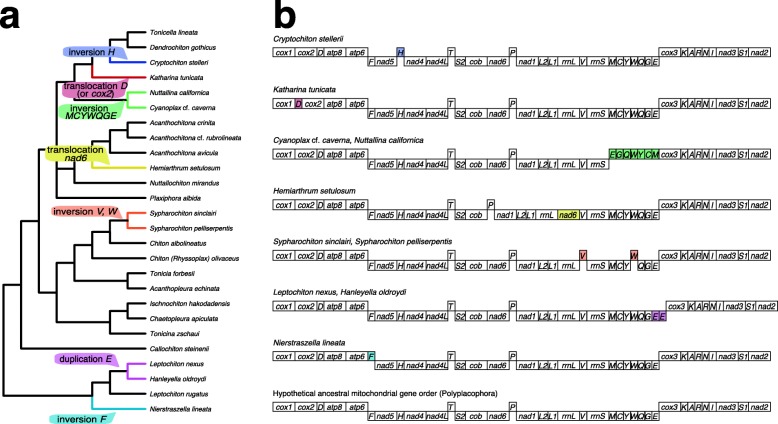
Evolution of mitochondrial gene order in chitons. **a** Hypothesized gene rearrangements mapped onto our Bayesian phylogeny (Additional file 4). **b** Described chiton mitochondrial gene orders. The hypothesized ancestral order for chitons is based on outgroup comparison and it is also the most frequent among chitons. Genes (not to scale) are depicted as encoded either by the major (upper) and minor (lower) strand and abbreviations follow Boore [39]. Rearranged genes are colored and their inferred origin is shown onto the phylogeny **a**

We newly reported the gene orders for 10 complete and 3 nearly complete mitogenomes, bringing the total number of chiton mitogenomes with fully or near-fully determined gene order to 22 (Fig. 1, Additional file 1). The new mitogenomes contained the same 37 genes that are typical for bilaterians [39], and mostly matched the gene order of *Chaetopleura apiculata* (Chitonina) that retains the inferred ancestral gene order for chitons [47]. Exceptions to this gene order were considered derived: (i) *Nierstraszella lineata* (Nierstrasz, 1905) (Lepidopleurida) displayed an inversion of the *trnF* gene, retaining its relative position but encoded on the major strand; (ii) *Hemiarthrum setulosum* (Acanthochitonina: Cryptoplacoidea) had a translocation in the *nad6* gene to a new position between the *rrnL* and *trnV* genes; and (iii) *Hanleyella oldroydi* and *Leptochiton nexus* (Lepidopleurida) contained two adjacent *trnE* genes (Fig. 1). We were unable to PCR amplify the region between the end of the *trnV* gene and the beginning of the *cox3* gene (that includes a putative control region) for *Nuttallochiton mirandus* (Thiele, 1906)*, Callochiton steinenii* (Pfeffer, 1886), and *Tonicina zschaui* (Pfeffer, 1886), and thus the relative gene order of the MCYWQGE tRNA cluster could not be fully determined (Additional file 1). For *Acanthopleura echinata* (Barnes, 1824) and *Tonicia forbesii* Carpenter, 1857 (Chitonina: Chitonidae), we were able to determine the gene order for this tRNA cluster but could not sequence the adjacent control region. For the three species with mitochondrial sequences derived from RNAseq data (*Leptochiton rugatus*, *Chiton* (*Rhyssoplax*) *olivaceus,* and *Tonicella lineata*) we explicitly avoided making claims about gene orders because the data proved insufficient to reconstruct intergenic regions with certainty.

### Mitogenomes helped resolving the chiton phylogeny

**Fig. 2:**
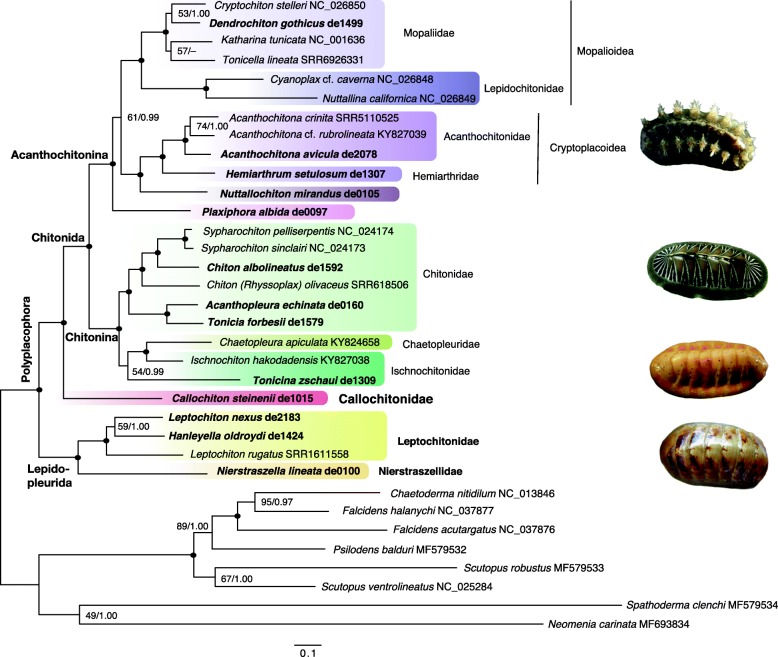
Mitogenomic phylogeny of chitons. Maximum likelihood phylogram inferred from the combined protein + rRNA dataset under the best-fit models and partitions (full tree available in Additional file 4). Numbers at nodes are respectively non-parametric bootstrap proportions (BP; %) and Bayesian posterior probabilities (BPP) from the maximum likelihood and Bayesian analyses, respectively (BI tree available in Additional file 4); dots represent maximum support (100/ 1.00). Scale bar is in expected substitutions site^− 1^. Higher taxonomic ranks are indicated and voucher (new mitogenomes; bold) or NCBI accession numbers are indicated for each species. Images (top to bottom): *Acanthochitona avicula, Chiton albolineatus, Callochiton steinenii,* and *Leptochiton rugatus*

Despite the ancient fossil history of chitons and the expected relatively rapid accumulation of substitutions in bilaterian mitogenomes, our inclusive analysis produced a result with robust statistical support for key relationships (Fig. 2). As rooted with aplacophorans, all our trees recovered a deep split within Polyplacophora between Lepidopleurida and Chitonida. Within Lepidopleurida, *Nierstraszella* (Nierstraszellidae) was the sister group to Leptochitonidae, which included *Hanleyella* and *Leptochiton,* the latter being recovered as paraphyletic. Within Chitonina, *Callochiton steinenii* (Callochitonidae) was the sister group of all remaining Chitonida, which comprises most extant chiton species diversity, split into Acanthochitonina and Chitonina (in this case excluding Callochitonidae).

Acanthochitonina contained three strongly-supported lineages (*Plaxiphora*, *Nuttallochiton* + Cryptoplacoidea, and Mopalioidea without *Plaxiphora* and *Nuttallochiton*) but their relative branching order was unresolved. In the ML and BI trees inferred from the combined dataset, as well as in the PhyloBayes MtZoa+Γ4 tree inferred from the protein dataset, *Plaxiphora* was resolved as sister to the other two lineages with variable support (0.99 BPP; ≤70% BP; Fig. 2 and Additional file 4). *Nuttallochiton* was recovered as sister to Cryptoplacoidea, including *Hemiarthrum* + three *Acanthochitona* spp., where *Acanthochitona crinita* and *Acanthochitona* cf. *rubrolineata* were sister taxa to the exclusion of *Acanthochitona avicula*, all relationships receiving strong support (Fig. 2). The monophyly of Mopaliidae sensu Kelly and Eernisse [25] was recovered with strong support, but the internal relationships were poorly resolved in both BI and ML trees (Fig. 2 and Additional file 4).

Within Chitonina, the trees based on combined matrices and the CAT-GTR BI tree favored *Tonicina zschaui* (Ischnochitonidae) as sister to *Chaetopleura apiculata* (Chaetopleuridae) + *Ischnochiton hakodadensis* (Ischnochitonidae), whereas *T. zschaui* was sister to all other members of Chitonina in BI and ML analyses of the protein matrix under MtZoa+F + I + Γ4 (0.99 BPP; ≤70% BP) (Fig. 2 and Additional file 4). Finally, *Chiton albolineatus*, *Chiton* (*Rhyssoplax*) *olivaceus* and *Sypharochiton* spp. were the sister group of *Acanthopleura* + *Tonicia* (Chitonidae), all relationships receiving strong statistical support (Fig. 2).

The small topological differences among the various analyses were not directly related to compositional differences among sequences. The amino acid composition of each species and results of compositional tests can be seen in Additional file 5. Compositional χ^2^-tests indicated that aplacophoran outgroups, as well as *Leptochiton rugatus* and *Callochiton steinenii* deviated most from the average composition. Pairwise matched-pair tests also indicated that most aplacophorans and the two chiton species mentioned above had the most deviant amino acid compositions, which resulted in non-stationary composition (evidenced by the high proportion of significant Stuart’s tests; Additional file 5). None of the mentioned species were involved in conflicting relationships in our trees.

### Molecular dating

Overall, the posterior ages estimated from the 24 experimental conditions (four calibration schemes, three prior fossil calibration densities, two clock models) were highly correlated (ρ > 0.92; Additional file 6). The largest differences among experimental conditions corresponded to using different fossil calibration distributions, with short-tailed t-cauchy distribution producing younger ages than long-tailed t-cauchy and uniform distributions (the latter two showed very similar ages; ρ > 0.96; Additional file 6). Short-tailed t-cauchy distributions represent strong priors that concentrate most of the prior probability close to fossil minima i.e., fossils ages are considered good proxies for the ages of the events being calibrated. Given the current knowledge of the chiton fossil record, such scenario might be unrealistic, and due to the large differences to other distributions, results from short-tailed t-cauchy analyses were disregarded in the following.

**Fig. 3:**
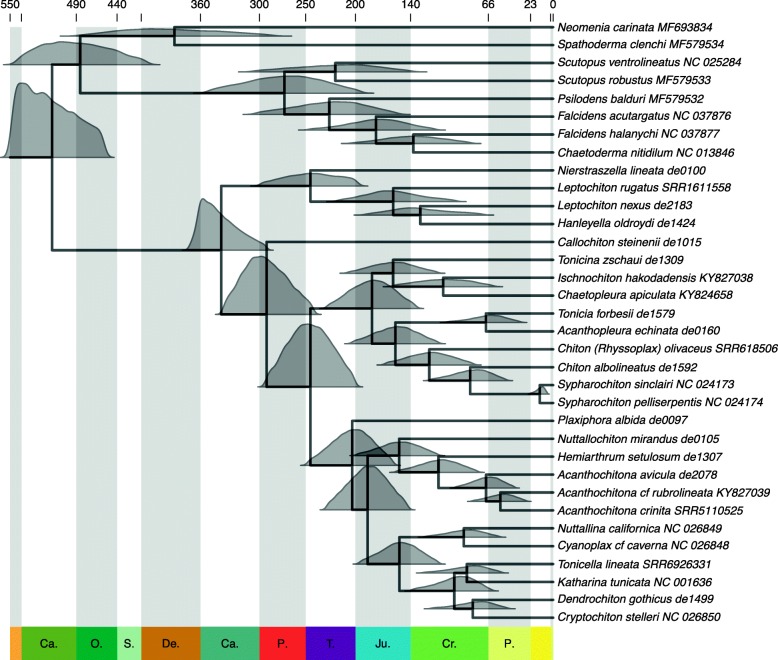
Time-calibrated phylogeny of Aculifera. Divergence times are inferred with MCMCTree under an uncorrelated relaxed clock and calibration Scheme 1 (fossils 1a-4a) using uniform calibrations. Node ages correspond to posterior means and full posterior distributions are also shown. Scale is in million years ago (Ma) and main geologic periods are highlighted

The estimated ages with long-tailed t-cauchy were similar to those using uniform distributions under the uncorrelated clock model assumption, whereas they produced comparatively older estimates when rate autocorrelation was assumed (Additional files 6 and 7). Long-tailed t-cauchy produced the widest 95% highest probability density (HPD) intervals across all experimental conditions. The second most important factor affecting the estimated ages was the molecular clock model. Assuming rate autocorrelation resulted in overall older estimates. The ages estimated under the two clock models were most different among long-tailed t-cauchy analyses, uniform analyses being less affected and producing ages more similar to those estimated under the uncorrelated clock model (Additional files 6 and 7). Finally, the use of alternative calibration schemes had the smallest effect (Additional files 6 and 7). Given these sensitivity analyses, the ages obtained under the uncorrelated molecular clock with calibration Scheme 1 (combination of 1a and 4a calibrations; see Material and Methods) and uniform distributions were the most stable and were thus used as the main analysis of reference, highlighting differences to other analyses when relevant (results from the 24 analytical conditions are available in Additional files 6 and 7). Moreover, uniform fossil calibrations are “flat priors” that are more appropriate in the absence of strong prior information from fossils. While several studies have argued that rate autocorrelation might be a more “biologically realistic” model, we obtained more stable estimates under the uncorrelated clock. Despite the minimal effect of different calibration schemes, Scheme 1 might represent the current best attempt of understanding the chiton fossil record (Fig. 3).

Assuming uncorrelated rates and uniform calibrations from Scheme 1 (Fig. 3), the crown group Polyplacophora was dated at 338 (95% HPD: 292–370) Ma in the Carboniferous, and the split between Callochitonidae and the remaining Chitonida at 292 (244–336) Ma in the Early Permian. The ages of Chitonida (excluding *Callochiton*) and Lepidopleurida were estimated at 247 (202–293) Ma and at 247 (198–289) Ma in the Triassic, respectively. The earliest divergences within Acanthochitonina (156–204 Ma) and within Chitonina (160–164 Ma) occurred approximately at the same time in the Jurassic period. The ancestor of Mopalioidea was estimated to occur 156 (166–197) Ma, whereas the families Mopaliidae and (part of) Lepidochitonidae (sensu [26]) were dated at 101 (63–144) and 91 (55–131) Ma, respectively.

## Discussion

### Utility of mitogenomes for resolving the chiton phylogeny

Compared to those of gastropods or bivalves, chiton mitochondrial genomes displayed a rather conserved gene order, most species retaining the inferred ancestral gene order for mollusks [7, 47], which in turn is conserved within Bilateria [37]. In chitons, the following rearrangements could be inferred: (i) an inversion of the *trnF* gene in at least *Nierstraszella lineata*; (ii) duplication of the *trnE* gene prior to the common ancestor of *Hanleyella* and *Leptochiton nexus*; (iii) inversions of the *trnV* and *trnW* genes before the common ancestor of both *Sypharochiton* species; (iv) translocation of the *nad6* gene in at least *Hemiarthrum setulosum*; (v) inversion of the MCYWQGE tRNA gene cluster prior to the common ancestor of *Cyanoplax* and *Nuttallina*; and (vi) translocation of the *trnD* gene (or the *cox2* gene) in at least *Katharina tunicata*. These rearrangements are inferred as derived by outgroup comparison to mitochondrial gene orders of other mollusks and bilaterians [7, 47]. Even though the relative gene order of the MCYWQGE tRNA cluster could not be fully determined in three species (*Nuttallochiton mirandus, Callochiton steinenii*, and *Tonicina zschaui*), it is likely that they conform to the ancestral order given their phylogenetic positions and the overall stasis in gene orders.

In agreement with the observation that tRNAs are often the most dynamic elements in mitogenomes [84], eight out of nine rearrangements involved exclusively tRNA genes. The tandem duplication and random loss model [85] is the most commonly invoked mechanism to explain gene rearrangements in mitogenomes [86]. This model could explain the transposition of the *nad6* gene in *Hemiarthrum*, the transposition of the *trnD* gene in *Katharina*, and the duplication of the *trnE* gene in *Leptochiton nexus* and *Henleyella*. In the latter case, the two *trnE* genes occurred in tandem and before a non-coding region that has been proposed to contain origins of replication and transcription similarly to the control region of chordates [87, 88], which has been shown to be a hotspot for gene rearrangement [86, 89]. Alternative mechanisms need to be invoked to explain the tRNA gene inversions in *Cyanoplax*, *Nuttallina*, *Cryptochiton*, and *Sypharochiton* spp., such as illegitimate recombination via minicircle [90]. Note that illegitimate recombination could also explain all the above-mentioned transpositions and duplications. The presence of any rare gene rearrangements in mitogenomes could each serve as an additional phylogenetic marker [40] that, for instance, could help clarifying the systematics within Acanthochitonina (tRNA gene rearrangements in *Nuttallina*, *Cyanoplax* and *Katharina*; [26]) or within Leptochitonidae (screening species for the duplication of the *trnE* gene found in *Hanleyella* and *Leptochiton nexus*).

Mitochondrial gene rearrangements have often been associated with increased evolutionary rates and compositional strand biases among species [37], which could confound phylogenetic inference methods. The fact that all protein-coding and rRNA genes are consistently encoded by the same strands in all sequenced chitons might have reduced the chance for rate and compositional heterogeneities among lineages. Less rearranged, slower evolving mitogenomes have been shown to produce more accurate phylogenies [46]. Despite the presence of non-stationary amino acid composition in our data (see Results), our phylogenetic analyses recovered fairly robust and congruent tree topologies, regardless of the applied models and inference methods, with only four unsettled branches left (Fig. 2, Additional file 4). All four instances are associated with short internal branches indicating potential radiation events, and these generally correspond to known taxonomic disagreements among available classification systems. Overall, mitogenomics stands out as a promising tool to clarifying the phylogeny of chitons. New chiton mitogenomes from yet unsampled lineages will likely produce robust phylogenies that resolve remaining controversies, reveal new ones, and ultimately improve our understanding of the chiton phylogeny. In addition, a phylogenomic exploration of diverse nuclear gene regions is expected to significantly contribute to this goal by providing an independent line of evidence to confirm or refute the mitogenomic phylogeny. Cost-effective high-throughput sequencing techniques such as transcriptomics and hybrid enrichment will permit broader taxon sampling and the high resolving power of (nuclear) phylogenomics, together with adequately accounting for systematic biases, will help resolve particularly difficult branching patterns, as demonstrated in other animal groups [4, 91].

### Chiton systematics, classification, and evolution

The deep structure of the chiton tree approximately corresponds with the currently recognized major lineages: a deep split separates the order Lepidopleurida from *Callochiton* and all other remaining Chitonida, the latter being divided into Chitonina and Acanthochitonina (Fig. 2). The position of Callochitonidae (represented here by *Callochiton*) has been a major point of controversy in chiton systematics [23, 27, 30, 92]. Our recovery of *Callochiton* as sister group to all other Chitonida agrees with several previous molecular studies [26, 93] but contradicts others. In Sigwart et al. [27] *Callochiton* was sister to Acanthochitonina, but this might be due to a limited representation of Chitonina and Lepidopleurida in their dataset. In Okusu et al. [24], *Callochiton* was sister to Lepidopleurida, a result that conflicts with morphological evidence and could derive from a combination of limited taxon and gene sampling (e.g. no *rrnL* data was available for *Callochiton*) and rooting problems. The position of *Callochiton* as sister to all other Chitonida is supported by its mostly smooth egg hull, symmetrically arranged mitochondria into an otherwise Chitonida-like sperm, and a fertilization process that has been characterized as “intermediate” between Lepidopleurida and all other studied Chitonida [32, 94]. In Lepidopleurida, fertilization occurs by fusion of sperm with a typical metazoan acrosome with the egg, thus transferring not only the chromatin but also the rest of the organelles into the egg cytoplasm, as is the case in most mollusks and metazoans [32]. In *Callochiton* and all other Chitonida, the sperm digests a minute pore in the egg hull and injects only the chromatin, leaving out all other organelles [32, 94]. This unique mechanism prevents the transmission of male mitochondrial DNA [1, 32]. Reports of dual mitochondrial inheritance are rare (but occur in some bivalve mollusks; 94) and no evidence for such mechanism exists in Lepidopleurida. Nevertheless, the fertilization in Lepidopleurida is known only for *Leptochiton asellus* plus indirect evidence from two other species [32] and a more general study with broader sampling of Lepidopleurida species would be needed to confirm the generality of these different fertilization processes. *Callochiton* has been included into Chitonida [23] based on the shared presence of slits in valve insertion plates and the typical lateral gill placement and not posterior as in Lepidopleurida [1, 33, 34, 96]. Without *Callochiton*, the remaining Chitonida could be defined by synapomorphies of asymmetrical sperm mitochondria [30] and the possession of elaborate egg hull projections [33, 34, 97], although egg hulls in Chitonina and Acanthochitonina are of two contrasting types and could have evolved independently.

Lepidopleurida are mostly defined based on plesiomorphic characters such as the presence of unslitted valve insertion plates, a posterior gill arrangement (adanal), simple gamete structures, and special aesthete innervation patterns [23, 96]. The only defining synapomorphy might be the sensory “Schwabe organ” [26]. From a molecular viewpoint the monophyly of extant Lepidopleurida has only been tested in a single study that included two genera of Lepidopleurida [28] as other analyses did not include non-chiton outgroups and assumed their monophyly (e.g., 28). The recognition of *Nierstraszella* in its own family Nierstraszellidae Sirenko, 1992 and away from representatives of Leptochitonidae is supported by its morphology, characterized by a fleshy proteinaceous layer that covers the dorsal shell surface [98]. Our analyses found *Leptochiton nexus* to be more closely related to *Hanleyella oldroydi* than to *Leptochiton rugatus*. Previous analyses of the species-rich cosmopolitan genus *Leptochiton* have not supported it as monophyletic, which has long been suspected given the vague anatomical diagnosis and the lack of defining synapomorphies [99].

In previous molecular phylogenies, the monophyly of Chitonina has been supported by Irisarri et al. [26] but not by Okusu et al. [24] due to the position of *Schizochiton incisus*, a hypothesis that could not be tested in our study. While the monophyly of the family Chitonidae was well supported, Ischnochitonidae was recovered as non-monophyletic, albeit with low support (*Ischnochiton hakodadensis* was closer to *Chaetopleura* than to *Tonicina*). About half of all living chiton species belong to Chitonina and resolving its phylogeny will require further studies with a broader taxon sampling.

Within Acanthochitonina, *Plaxiphora* was recovered either as sister to all other Acanthochitonina (partitioned analyses of the combined matrices; 0.99 BPP and < 70% BP; Fig. 2 and Additional file 4) or as sister to Mopalioidea to the exclusion to Cryptoplacoidea (BI CAT-GTR, 0.68 BPP and ML MtZoa, 42% BP; Additional file 4). In either case, *Plaxiphora* lies well outside Mopaliidae, as shown previously by other molecular studies [26, 27]. This is in agreement with aesthete morphology: *Plaxiphora* shows more similarities to Acanthochitonidae than to Mopaliidae [29]. The large phylogenetic distance between *Plaxiphora* and *Mopalia* (Mopaliidae) is noteworthy given their similarities in external morphology, with a broad body outline and girdles covered with corneous hairs (*Plaxiphora*) or setae (*Mopalia* and other members of Mopaliidae). If this represents a case of convergence, as hypothesized previously for other chitons [23], the adaptive advantages of this morphology are worth investigating. One of such similarities between *Plaxiphora* and *Mopalia* is the presence of a sinus in the posterior valve, long used as a defining character for Mopaliidae [23, 100]. However, our topology implies that the posterior sinus probably evolved multiple times independently in Acanthochitonina, which has been suggested to associate with escalating demands of oxygen in response to increasing body size [26]. *Nuttallochiton* was found to be closely related to other included Cryptoplacoidea with strong support, in agreement with previous molecular studies [24, 27]. This implies that the current taxonomic placement of *Nuttallochiton* within Mopaliidae [23] is likewise in need of revision. Our analyses, in agreement with the latest molecular studies, confirm the inclusion of *Hemiarthrum* in Cryptoplacoidea [26, 27], supported by the presence of spicule tufts in its girdle and abanal gill features [92].

The Mopaliodea grouping of Mopaliidae plus Lepidochitonidae, each as currently defined [26], was strongly supported as in previous studies [26, 27]. The inclusion of members of *Tonicella* as nested within Mopaliidae (e.g. [25], herein) precludes the alternative association of genera here grouped as Lepidochitonidae Iredale, 1914 within Tonicellidae Simroth, 1894 [23], although we point out that the latter could be a senior synonym of Mopaliidae Dall, 1889 with further study. Meanwhile, Mopaliidae comprises morphologically diverse genera that were formerly placed in other families, united by their mostly North Pacific distribution [25, 77]. In contrast, *Nuttallochiton* and *Plaxiphora,* conventionally members of Mopaliidae, occur mostly in the Southern Hemisphere [101, 102]. The geographic restriction of the North Pacific clade thus has few exceptions, such as the North Atlantic *Boreochiton ruber* (Linnaeus, 1767) and *Tonicella marmorea* (O. Fabricius, 1780), but these species are either the same or very similar species within these genera of otherwise exclusively North Pacific distribution [103], which suggests a geologically recent invasion of the North Atlantic. The only other exception is *Placiphorella*, whose deep-water members have a nearly cosmopolitan distribution [104].

### A molecular timescale for chiton evolution

According to paleontological and embryological studies, a Cambrian [105] chiton-like aculiferan ancestor with seven or eight dorsal plates [8, 11] gave rise to living chitons (Neoloricata), Solenogastres and Caudofoveata (which underwent a secondary simplification towards their current vermiform morphology) and other fossil forms including “paleoloricates” and multiplacophorans [6, 9, 106]. The Cambrian split between extant chitons, solenogasters, and caudofoveates is recovered by our timetrees, regardless of whether Ordovician (*Echinochiton*; calibration 1a) or Silurian (*Acaenoplax*; calibration 1b) fossils were used to calibrate this node, and in agreement with previous molecular clock analyses [6, 11]. Our molecular clock analyses inferred a Carboniferous age for the crown-group Polyplacophora, regardless of whether Early Jurassic *Allochiton, Heterochiton, and Ischnochiton marloffsteinensis* (calibration 4a) or more recent Late Cretaceous *Chiton* (calibration 4b) were used as calibrations for the first appearance of Chitonida. The Carboniferous age of the crown-group Polyplacophora agrees not only with previous molecular clock analyses [6, 11] but also with the fossil record, where the earliest neoloricates with an articulamentum shell layer extending as sutural laminae (or apophyses) are found in Carboniferous deposits [3, 23]. Note however that this shell layer has been reported in multiplacophorans [107]. The articulamentum shell layer would eventually provide new opportunities of increased complexity in both musculature binding of valves and to the girdle, likely resulting in greater mobility [23]. The Late Carboniferous to Early Permian age estimated for Chitonida (excluding *Callochiton*) is in line with previous molecular clock analyses [6, 11] and some fossils. However, there remains much uncertainty about the interpretation of Paleozoic fossils. Notably, the phylogenetic affinity of the Permian *Ochmazochiton comptus* Hoare & Smith, 1984 has been debated, being considered either within (e.g., [23]) or outside (e.g., [6]) the Chitonida crown group, which has implications for the first appearance of Chitonida. Sirenko [23] interpreted the jagged margins of insertion plates as primitive slits that might have functioned, as in extant Chitonida, to allow the innervation of the dorsal tegmentum sensory organs (aesthetes), but these slits show very little resemblance to the slit rays of extant Chitonida.

The origin of crown-group Lepidopleurida was dated in the Triassic. The inferred Jurassic age of the family Leptochitonidae is somewhat younger than the earliest records of fossils identified as *Leptochiton* in the Late Triassic [20]. This disagreement could indicate problems due to limited taxon sampling, misspecified molecular clock models, or uncertainties in available calibrations. Alternatively, a Jurassic age of Leptochitonidae would imply that older fossils could be currently misclassified within the family and thus in need of a careful re-examination.

Interestingly, the chronology of events described above for the deepest splits within Aculifera and Polyplacophora agree with some previous molecular clock analyses that differed substantially from ours in taxon sampling and methodology: a metazoan-wide molecular dataset with limited chiton representatives calibrated with fossils exclusively outside of Polyplacophora [6] or the same dataset complemented with morphological characters of extant and extinct species into a total-evidence analysis [11]. However, this apparent agreement might also derive in part by the use of relatively broad (conservative) calibrations in our analyses, reflecting the inherent uncertainty associated with Paleozoic aculiferan fossils. Fossil calibrations are often the most crucial aspect in molecular clock analyses and *a priori* paleontological evaluation of calibrations remains the best strategy to ensure accurate molecular dates [108]. Moreover, the precision of estimated divergence times (HPD intervals) also reflects the uncertainty underlying the fossil record [81] and improving such estimates will necessarily require better knowledge of the molluscan paleontological record.

According to our timetrees, the early divergences within Chitonina occurred in the Jurassic, followed by divergences of most families and subfamilies represented in our timetree during the Cretaceous. This includes members of Chitonidae that represent, together with *Lorica* (Loricidae), the oldest known fossils for extant taxa within Chitonina [23, 79]. In this case, the Jurassic *Allochiton* and *Heterochiton* [76], earlier assigned to Mopaliidae (Acanthochitonina) (e.g., 23) but recently treated more generally as early Chitonida [20], and *Ischnochiton marloffsteinensis* would represent some of the oldest known members of Chitonida. Awaiting a careful re-evaluation of characters in these fossils (such as the presence of slit rays)*,* our inferred Triassic to Jurassic ages are potentially compatible with their classification within Acanthochitonina (*Allochiton, Heterochiton*) and Chitonina (*Ischnochiton marloffsteinensis*) (Fig. 3).

Members of Mopaliidae and part of Lepidochitonidae as currently defined [25, 26], display a mostly North Pacific distribution. Several of its genera have been hypothesized to diversify in the last 16 Ma, after the Late Miocene cooling of the North Pacific, possibly mediated by an increase in productivity and environmental heterogeneity [25, 77]. The fossil record shows a high diversity of chiton species, including members of Mopaliidae, in the Pacific coast by the Late Pliocene, but chitons are strikingly absent from the known Miocene deposits of Western North America [23, 25, 77]. Our molecular clock analyses inferred Cretaceous ages for the common ancestors of both Lepidochitonidae and Mopallidae (92 and 104 Ma, respectively, under our preferred analysis; Fig. 3). These older estimates seem to be in conflict with the hypothesized Late Miocene diversification within *Mopalia* [25], but our taxon sampling and the lack of older fossils does not currently allow testing the deeper diversification within Mopaliidae, Lepidochitonidae, and Mopalioidea as a whole. As a consequence, we call for a more focused study with appropriate taxon sampling and combining molecular clocks and biogeographic reconstructions.

## Conclusions

We demonstrate the suitability of mitogenomes to infer robust molecular phylogenies of living chitons. We find an overall stasis in chiton mitochondrial gene orders, which may be beneficial for phylogenetic reconstruction by limiting the negative effects of rate and compositional heterogeneity among lineages. In addition, the rare genomic reorganizations involving mostly tRNA genes may be seen as molecular synapomorphies with taxonomic value. The inferred phylogenetic tree largely agrees with the latest advances in chiton phylogeny and taxonomy, but also reveal important changes that call for a revision of the higher-level classification of chitons. Moreover, the proposed phylogenetic hypotheses shed light into the evolution of several morphological characters, identifying new instances of convergence in external morphology. In this sense, our study illustrates the importance of considering independent data sources (e.g., from molecules and morphology) to better understand the origin and evolution of morphological characters and assess their phylogenetic and taxonomic utility. The divergences inferred by our molecular clock analsyes largely agreed with previous timetree estimates and the fossil record, but there remains considerable uncertainty associated with available fossil calibrations. In the near future, nuclear phylogenomic and emerging mitogenomic datasets are expected to significantly advance the resolution of the chiton phylogeny.

## Supplementary information

**Additional file 1.** Taxon sampling, locality and specimen vouches. Information on taxon sampling, locality and specimen vouchers.

**Additional file 2.** PCR primers and mitogenome annotation. Information on universal and species-specific PCR primers and annotation of newly sequenced mitogenomes.

**Additional file 3.** Model selection, model cross validation, and convergence of Bayesian analyses. Selection of best-fit models and partitions (IQ-TREE), model cross-validation (PhyloBayes), and convergence of Bayesian analyses (PhyloBayes, MrBayes).

**Additional file 4.** Additional phylogenetic trees. Additional results from partitioned and unpartitioned maximum likelihood and Bayesian analyses.

**Additional file 5.** Compositional heterogeneity. Results from χ^2^-tests (IQ-TREE) and matched-pair tests of symmetry (SymTest) from the protein dataset.

**Additional file 6.** Time-calibrated trees and correlation coefficients. Time-calibrated trees from all 24 experimental conditions (MCMCTree) and matrix of correlation coefficients among mean posterior ages.

**Additional file 7.** Inferred divergence times. Inferred mean ages and 95% highest probability density (HPD) for all 24 experimental conditions (MCMCTree) and reference tree with node IDs.

## Data Availability

The datasets supporting the conclusions of this article are available as Additional files and in the Figshare repository, https://doi.org/10.6084/m9.figshare.7963712.

## Abbreviations

BI: Bayesian inference
BIC: Bayesian information criterion
BP: Bootstrap proportions
BPP: Bayesian posterior probabilities
HPD: Highest probability density
Ma: Million years ago
ML: Maximum likelihood
rRNA: Ribosomal RNA
t-cauchy: Truncated cauchy
tRNA: Transfer RNA
