# Supplementary material for: A mitogenomic phylogeny of chitons (Mollusca: Polyplacophora)
Source: BMC Evol Biol. 2020 Feb 5;20:22. doi: 10.1186/s12862-019-1573-2 (PMC7003433; doi:10.1186/s12862-019-1573-2)
Supplement: Supplementary file 4 — Additional file 4. Additional phylogenetic trees. Additional results from partitioned and unpartitioned maximum likelihood and Bayesian analyses. [file 12862_2019_1573_MOESM4_ESM.pdf]

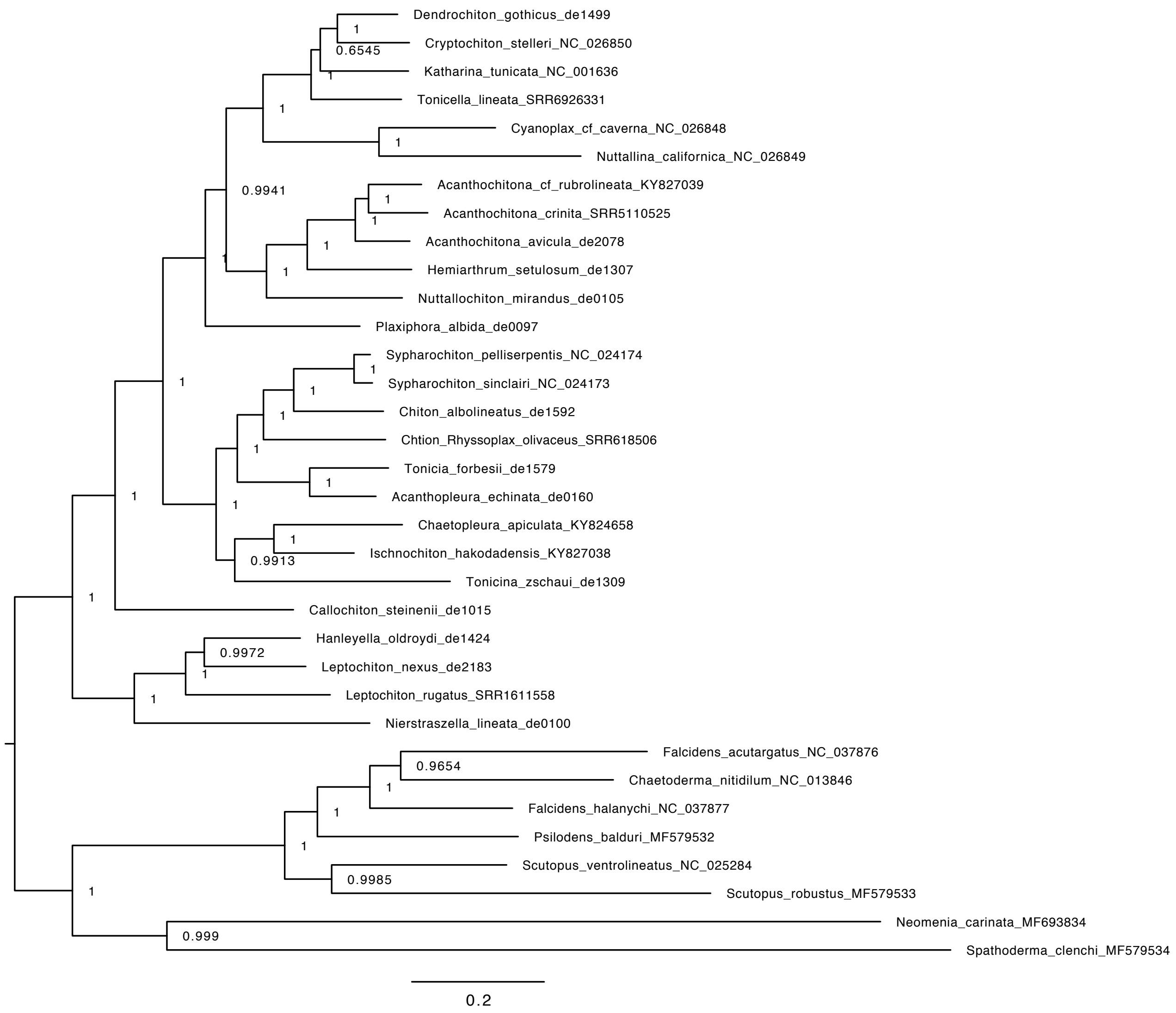

Partitioned Bayesian phylogram inferred from the combined (protein + rRNA) dataset in MrBayes. Numbers at nodes are posterior probabilities and scale bar is in expected substitutions site^{-1}.

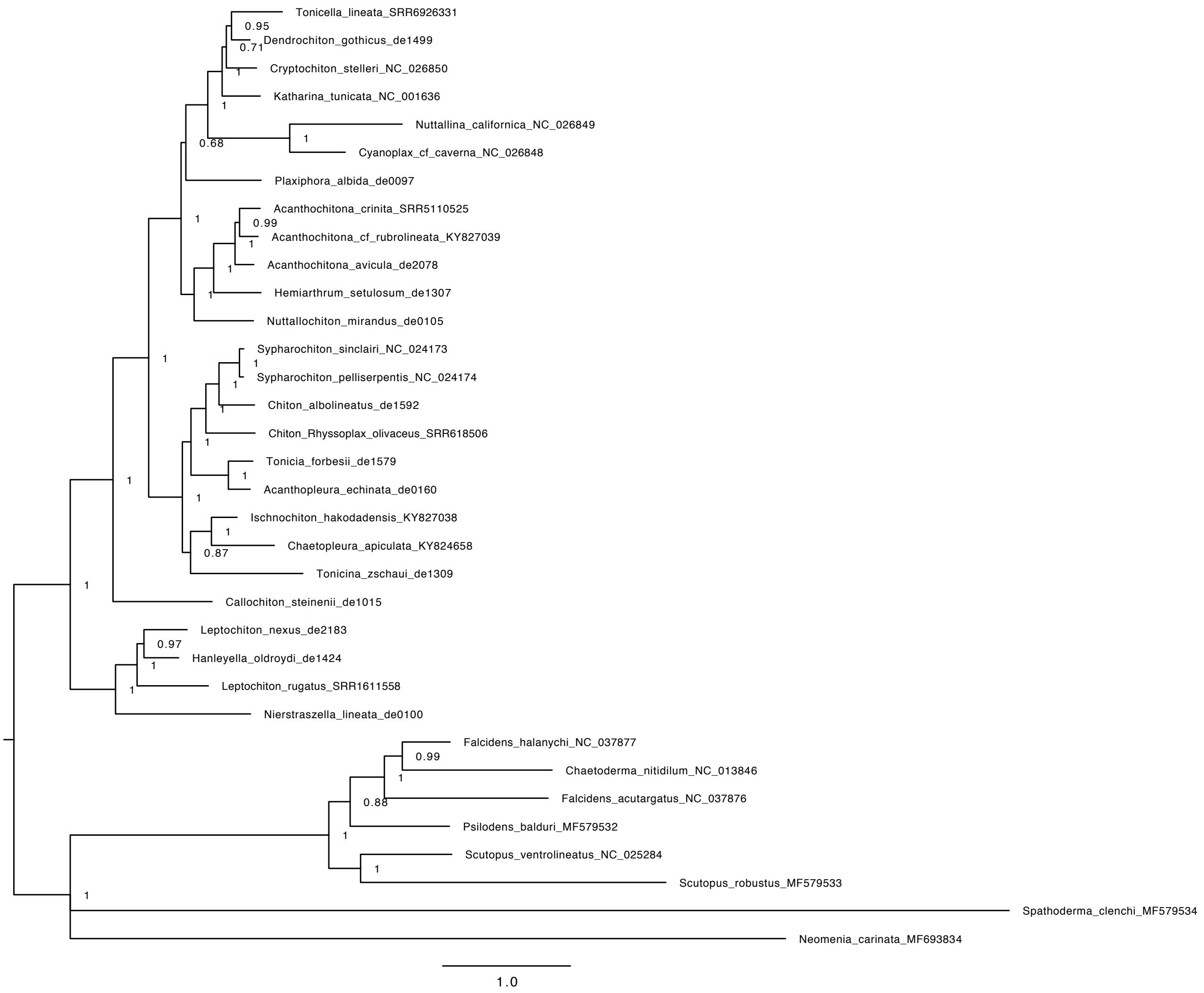

Bayesian phylogram inferred from the protein dataset under the CAT-GTR+Γ4 model in PhyloBayes. Numbers at nodes are posterior probabilites and scale bar is in expected substitutions site^{-1}.

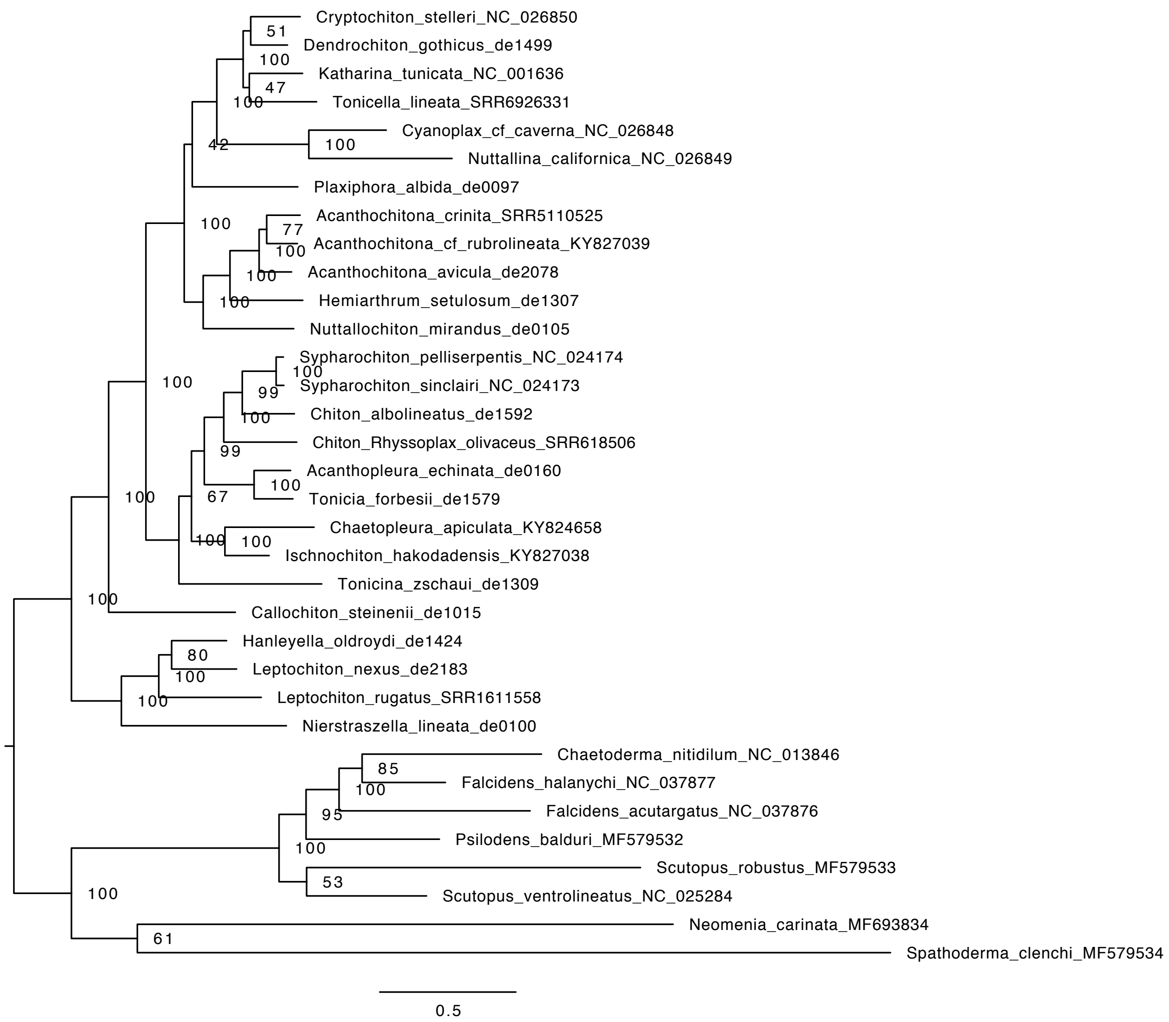

Maximum likelihood phylogram inferred from the protein dataset under the mtZOA+F+I+Γ4 model in IQTREE. Numbers at nodes are non-parametric bootstrap proportions (%) and scale bar is in expected substitutions site^{-1}.

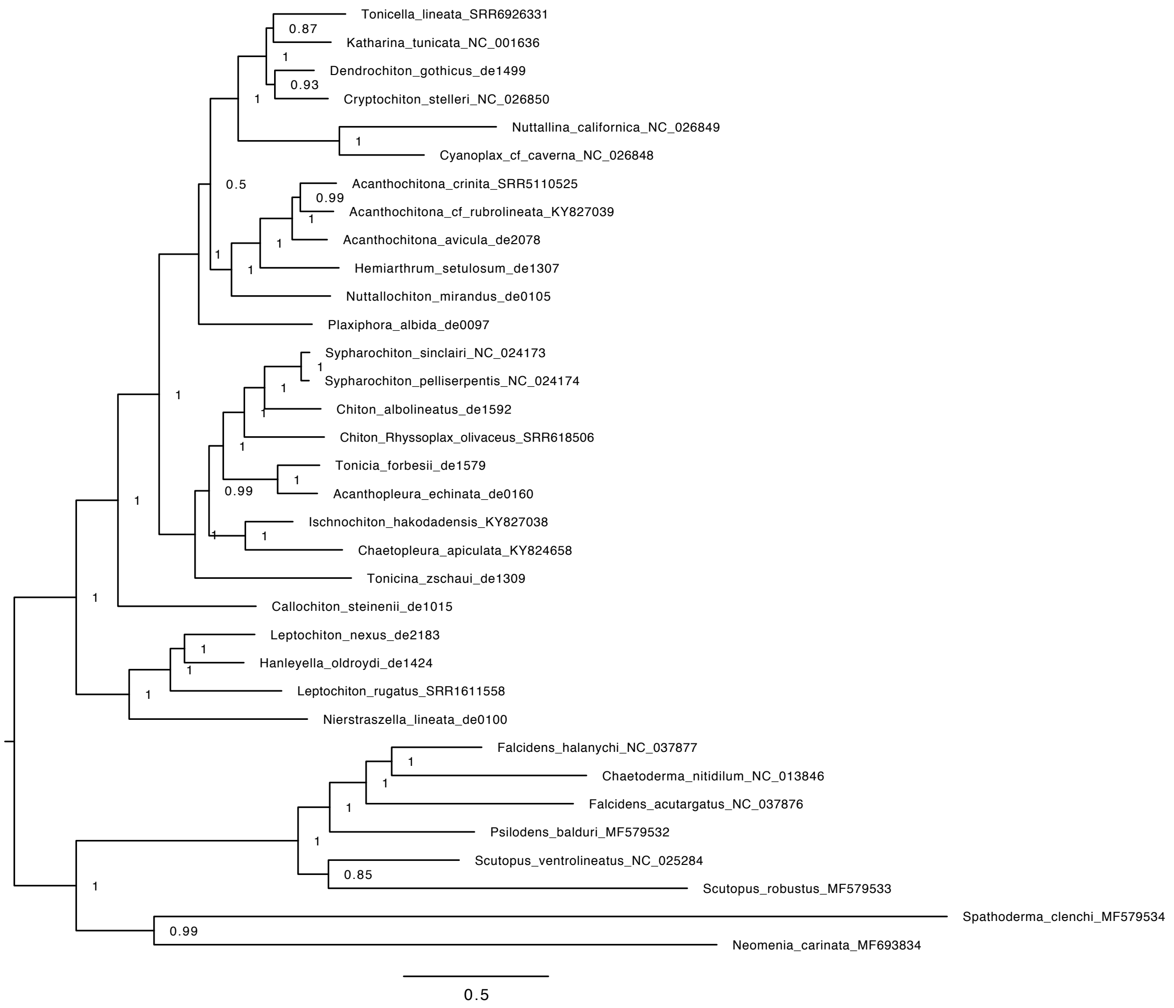

Bayesian phylogram inferred from the protein dataset under the mtZOA+Γ4 model in PhyloBayes. Numbers at nodes are posterior probabilites and scale bar is in expected substitutions site^{-1}.
