# Supplementary material for: A mitogenomic phylogeny of chitons (Mollusca: Polyplacophora)
Source: BMC Evol Biol. 2020 Feb 5;20:22. doi: 10.1186/s12862-019-1573-2 (PMC7003433; doi:10.1186/s12862-019-1573-2)

Supplementary File 6: Results of divergence time estimation

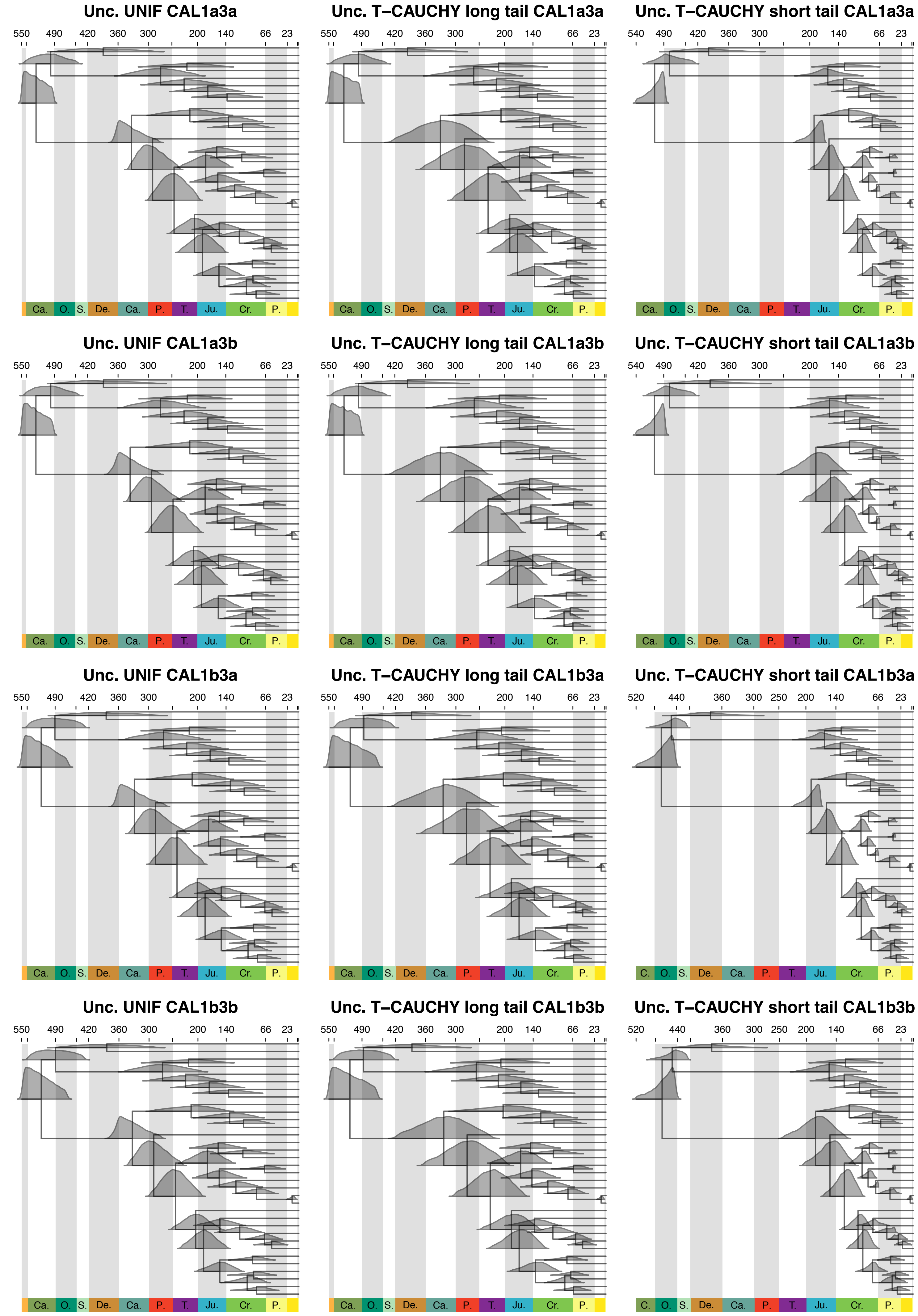

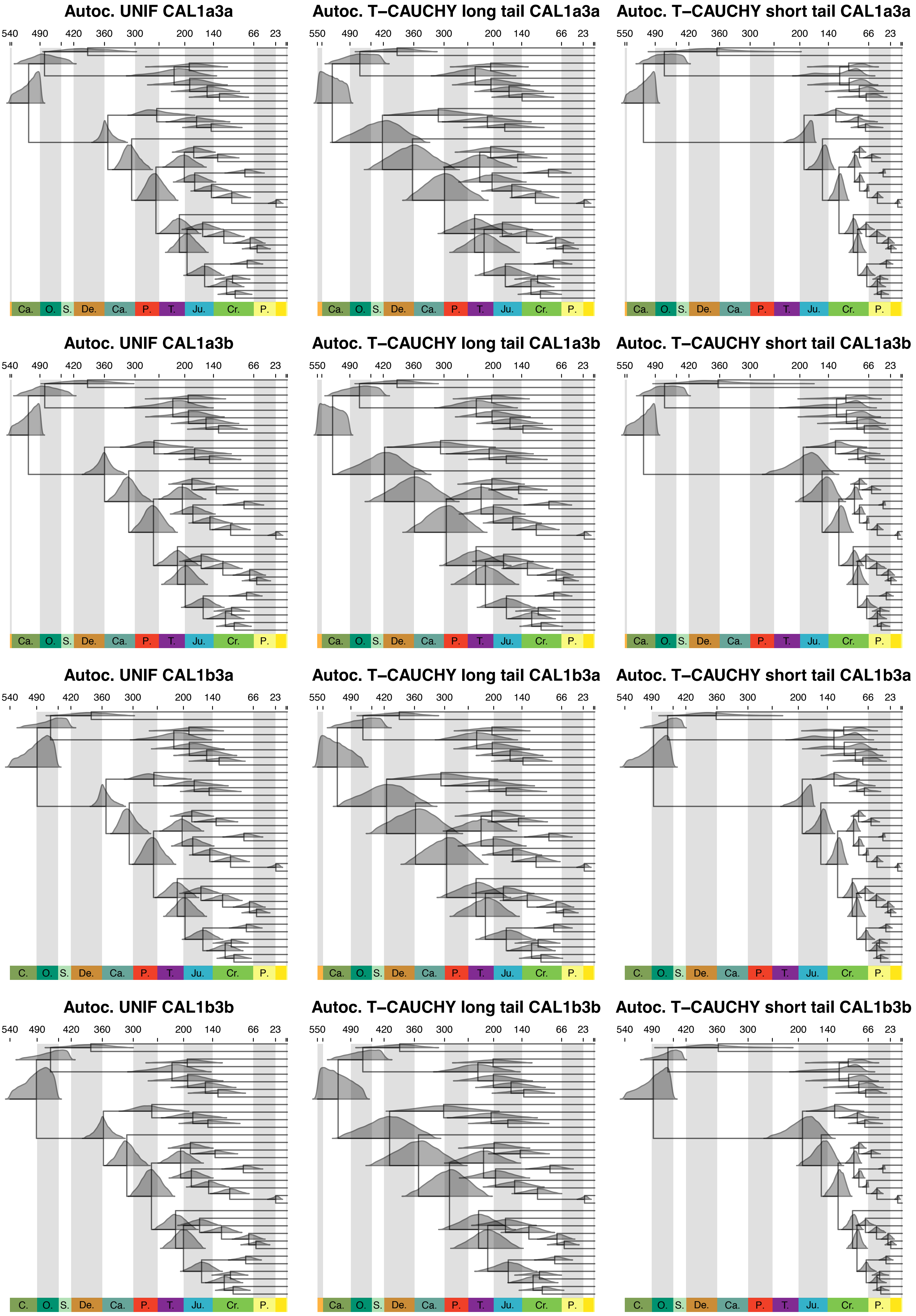
Autoc. UNIF CAL1a3a
540 490 420 360 300 200 140 66 23
Ca. O. S. De. Ca. P. T. Ju. Cr. P.
Autoc. T−CAUCHY long tail CAL1a3a
550 490 420 360 300 200 140 66 23
Ca. O. S. De. Ca. P. T. Ju. Cr. P.
Autoc. T−CAUCHY short tail CAL1a3a
550 490 420 360 300 200 140 66 23
Ca. O. S. De. Ca. P. T. Ju. Cr. P.
Autoc. UNIF CAL1a3b
540 490 420 360 300 200 140 66 23
Ca. O. S. De. Ca. P. T. Ju. Cr. P.
Autoc. T−CAUCHY long tail CAL1a3b
550 490 420 360 300 200 140 66 23
Ca. O. S. De. Ca. P. T. Ju. Cr. P.
Autoc. T−CAUCHY short tail CAL1a3b
550 490 420 360 300 200 140 66 23
Ca. O. S. De. Ca. P. T. Ju. Cr. P.
Autoc. UNIF CAL1b3a
540 490 420 360 300 200 140 66 23
C. O. S. De. Ca. P. T. Ju. Cr. P.
Autoc. T−CAUCHY long tail CAL1b3a
550 490 420 360 300 200 140 66 23
Ca. O. S. De. Ca. P. T. Ju. Cr. P.
Autoc. T−CAUCHY short tail CAL1b3a
540 490 420 360 300 200 140 66 23
C. O. S. De. Ca. P. T. Ju. Cr. P.
Autoc. UNIF CAL1b3b
540 490 420 360 300 200 140 66 23
C. O. S. De. Ca. P. T. Ju. Cr. P.
Autoc. T−CAUCHY long tail CAL1b3b
550 490 420 360 300 200 140 66 23
Ca. O. S. De. Ca. P. T. Ju. Cr. P.
Autoc. T−CAUCHY short tail CAL1b3b
540 490 420 360 300 200 140 66 23
C. O. S. De. Ca. P. T. Ju. Cr. P.

Supplementary File 6: Correlation coefficient of mean divergence times across analyses

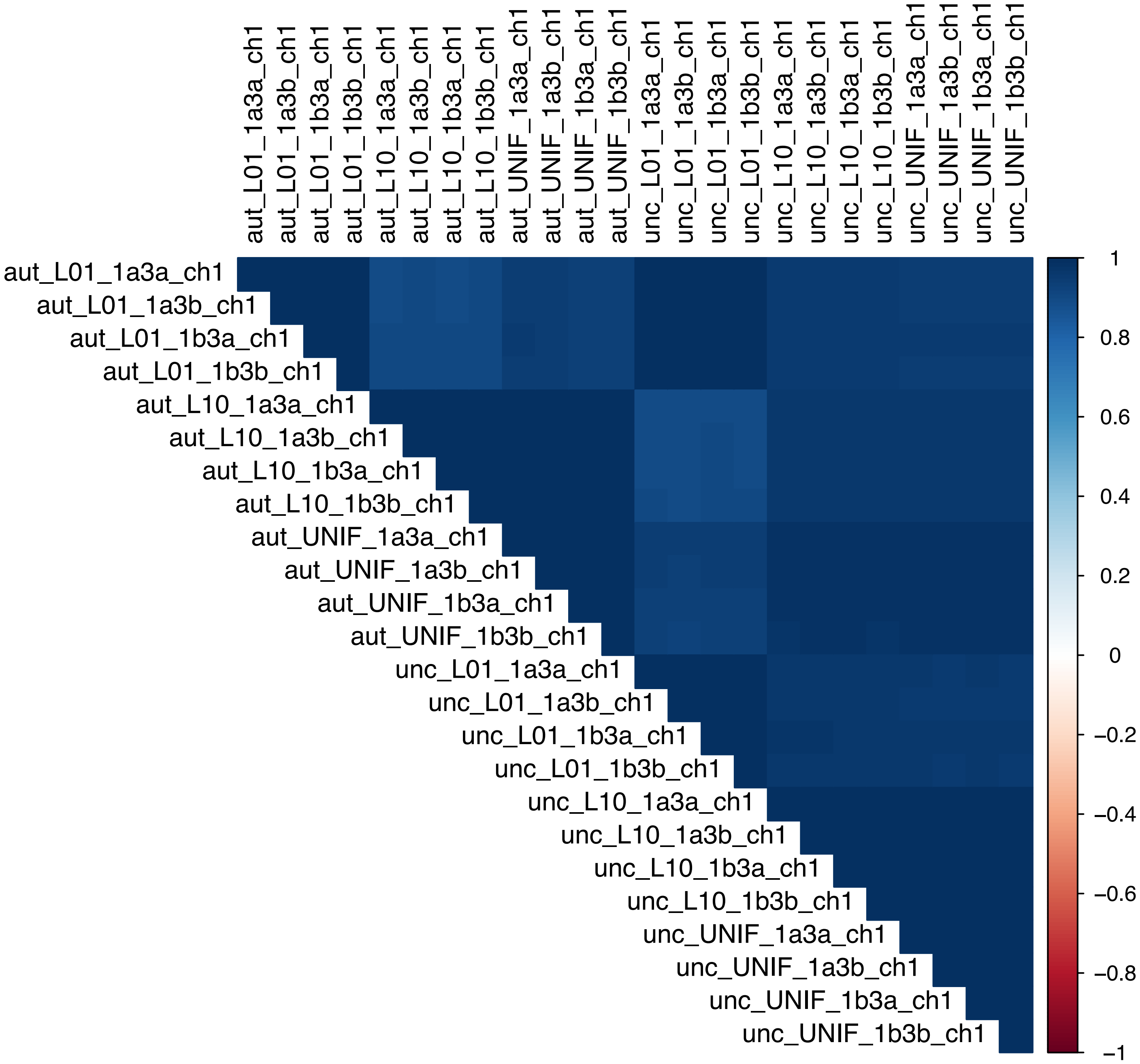

Supplement: Supplementary file 6 — Additional file 6. Time-calibrated trees and correlation coefficients. Time-calibrated trees from all 24 experimental conditions (MCMCTree) and matrix of correlation coefficients among mean posterior ages. [file 12862_2019_1573_MOESM6_ESM.pdf]
